# Cold-Blooded Attention: Finger Temperature Predicts Attentional Performance

**DOI:** 10.3389/fnhum.2017.00454

**Published:** 2017-09-12

**Authors:** Rodrigo C. Vergara, Cristóbal Moënne-Loccoz, Pedro E. Maldonado

**Affiliations:** ^1^Departmento de Neurociencia & Biomedical Neuroscience Institute, Facultad de Medicina, Universidad de Chile Santiago, Chile; ^2^Departamento de Ciencias de la Computación, Escuela de Ingeniería, Pontificia Universidad Católica de Chile Santiago, Chile

**Keywords:** autonomic response, cognition, body temperature, attention, workload

## Abstract

Thermal stress has been shown to increase the chances of unsafe behavior during industrial and driving performances due to reductions in mental and attentional resources. Nonetheless, establishing appropriate safety standards regarding environmental temperature has been a major problem, as modulations are also be affected by the task type, complexity, workload, duration, and previous experience with the task. To bypass this attentional and thermoregulatory problem, we focused on the body rather than environmental temperature. Specifically, we measured tympanic, forehead, finger and environmental temperatures accompanied by a battery of attentional tasks. We considered a 10 min baseline period wherein subjects were instructed to sit and relax, followed by three attentional tasks: a continuous performance task (CPT), a flanker task (FT) and a counting task (CT). Using multiple linear regression models, we evaluated which variable(s) were the best predictors of performance. The results showed a decrement in finger temperature due to instruction and task engagement that was absent when the subject was instructed to relax. No changes were observed in tympanic or forehead temperatures, while the environmental temperature remained almost constant for each subject. Specifically, the magnitude of the change in finger temperature was the best predictor of performance in all three attentional tasks. The results presented here suggest that finger temperature can be used as a predictor of alertness, as it predicted performance in attentional tasks better than environmental temperature. These findings strongly support that peripheral temperature can be used as a tool to prevent unsafe behaviors and accidents.

## Introduction

Environmental temperature has an important impact on behavior. For instance, thermal stress increases the chances of unsafe behavior during industrial work (Ramsey et al., 1983), impairing cognitive functions (Mazloumi et al., 2014), as well as diminishing driving performance (Hancock, 1986). Thermal stress has been proposed to drain attentional resources (Vasmatzidis et al., 2002; Hancock and Vasmatzidis, 2003; Cheema and Patrick, 2012; Liu et al., 2013; López-Sánchez and Hancock, 2017), therefore reducing task performance and increasing the chance of accidents. Nonetheless, configuring appropriate safety standards regarding environmental temperature has been a major problem, as modulations in task performance also involve the task type, complexity, workload, duration and previous experience with the task (Hancock, 1986; Pilcher et al., 2002; Hancock and Vasmatzidis, 2003; Gaoua et al., 2011). Even more importantly, these difficulties reduce the chances of suggesting an optimal environmental temperature during work instead of advising harmless ranges.

The key to understanding the wide range of tasks affected by environmental temperature should be rooted in an organism physiological mechanism. Nevertheless, despite the important bulk of literature regarding this phenomenon, no physiological explanation for this process exist other than considering environmental temperature as another stress factor (Hancock et al., 2007). Furthermore, many reports present the effects of environmental temperatures within the thermal comfort zone (≈21–24°C; Hancock, 1986; Pilcher et al., 2002; Steinmetz and Mussweiler, 2011; Cheema and Patrick, 2012; Huang et al., 2014; Schilder et al., 2014), supporting that this effect goes beyond thermal stress. In fact, electroencephalogram (EEG) attentional event-related potentials (ERP) during a decision making task modulates within the thermoneutral zone (Vergara, 2015). Therefore, these behavioral modulations putatively rooted on attentional phenomena have, in fact, neural correlates supporting thermal stress as an attentional phenomenon. We still lack understanding of a precise physiological mechanism explaining why environmental temperature can modulate behavior, and to a lesser extent, within a range that should be stressless.

Interestingly, most previous studies have focused on environmental temperature rather than thermoregulation physiology. Temperature itself plays a critical role in physiology that should be considered when suggesting it as a stressor. Because warm-blooded organisms must control their internal temperature within a small range, we might expect that their internal physiology would be in equilibrium with environmental temperature. This equilibrium is achieved by autonomic modulations that produce peripheral vasodilation/vasoconstriction to increase/decrease skin thermal conductivity, thus also increasing/decreasing skin temperature (Romanovsky, 2014). As such, the human body can regulate how much heat dissipates into the environment. When skin blood flow (mainly in the limbs) is not enough to couple with the environmental temperature and associated conditions (such as wind and humidity), sweating occurs if the temperature is too hot, and an increase in thermogenesis (internal heat production in brown adipose tissue) and shivering occur if the temperature is too cold (for more details see Romanovsky, 2007).

This mechanism suggests that humans can find physiological modulations for any change in environmental temperature, even within the non-stress range. Despite the fact that humans have an efficient homeostatic system that maintains body temperature within a small range of variation (Satinoff, 1978), it would be highly adaptive for warm-blooded animals increasing or reducing attention by means of alertness according to the environmental temperature to better thermoregulate behaviorally (Flouris, 2011; Terrien et al., 2011).

As such, we believe that attentional modulations due to environmental temperature should be better captured by variables related to thermoregulation physiology rather than environmental temperature itself. This approach should overcome time of exposure problems as well as subject variability using the surface to volume ratio, the amount of fat tissue, the clothing, or the task difficulty. If thermoregulatory processes do in fact affect attentional performance, overcoming these problems by measuring a biomarker of thermoregulation would essentially lead to a biomarker of attentional performance.

In the present work, we focused on using peripheral biomarkers of thermoregulatory processes to predict attentional states. Specifically, we used the core body temperature (tympanic temperature), the central border temperature (forehead temperature), the peripheral border temperature (fingertip temperature) and the environmental temperature (room temperature) to predict performance in three different attentional tasks. These tasks were meant to measure sustained attention, resilience to distractors, and attentional resources. We hypothesized that body temperature rather than environmental temperature would predict performance in attentional tasks and thus constitute an appropriate predictor of task competence.

## Materials and Methods

### Participants

Volunteer students from the Universidad de Chile were recruited by publicly posting the research invitation on social media. Students volunteered themselves by contacting us via e-mail. Informed consent was given in advance, and all students willing to participate were recruited. A total of 19 participants (8 females and 11 males) of ages ranging from 19 to 36 years old were recruited, with a mean age of 25.3 ± 5.5 years (mean ± SD). All participants reported normal or corrected-to-normal vision and no background of neurological or psychiatric conditions. The sample size used in this study was estimated based on the effect size of preliminary results. This study was approved by the Ethics Committee for Research in Humans from the Faculty of Medicine at the University of Chile with project number ID 060-2015, ACTA AP-65. All subjects gave written informed consent following the Declaration of Helsinki.

### Tasks

The participants executed one control task and three cognitive tasks during a single working session, a baseline, a continuous performance task (CPT), a flanker task (FT) and a counting task (CT), lasting approximately 10 min each. The baseline task (BT) was aimed to measure the baseline body temperature variation. In this task, participants were seated in front of a screen in the same fashion as if they were to execute any of the other tasks for approximately 10 min, which was the time estimated for each of the remaining tasks. We instructed the subject to “Sit down looking at the screen and relax. Please do not to fall asleep, and try not to close your eyes for long periods of time”. This instruction was given to achieve a relaxed but aware state.

#### CPT Task

**Figure 1:**
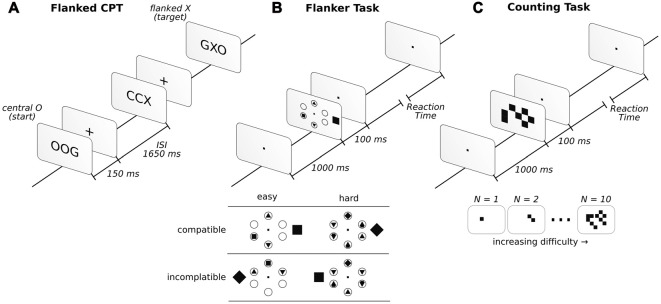
Description of the three attentional tasks in the order in which they were performed. Before starting the attentional tasks, a baseline was taken under the instruction “sit and relax” (for more details, see “Materials and Methods” Section). In the flanked continuous performance task (CPT; **A**), participants had to report whether the sequence of central letters O-any letter-X appeared. In the flanker task (FT; **B**), participants had to report a square or a diamond inside one of six circles. If the inside target figure (square or diamond) matched the external figure presented outside the circles, it was considered compatible, while those that did not match were incompatible. In the counting task (CT; **C**), participants had to report the number of squares observed.

A common version of this test consists of the detection of a letter with a low frequency of appearance (approximately 10%). However, many other versions exist wherein a sequence of letters is targeted rather than a single letter (Riccio et al., 2002; Huang-Pollock et al., 2012). To prevent the ceiling effect, we selected one of the most difficult versions of this task. In doing so, we flanked the target letter with an additional letter to the right and left of the central letter, thus making the task to detect a sequence of letters across the trial. This sequence was specifically a 2-back task. As such, we displayed three letters at the same time for 150 ms, followed by a fixation cross for 1650 ms. If the central letter was the letter X and, two trials back, the central letter was an O, participants reported by pressing a button (Go condition). Reports of seeing this sequence when it was not present were considered false alarms (False Alarm condition). A description of the task can be found in Figure 1A. The Go condition was randomly displayed in 15% of 400 trials in total, and the letters employed for the displays included only C, G, O, Q, H and X. All participants had to detect the same letter sequence.

#### Flanker Task

The FT was configured after that of Green and Bavelier (2003). In this task, six circles were arranged equidistance apart (2.1°) from the center of the screen in a circular fashion. In each trial, a distractor outside the circular array was presented. The distractor could have the shape of a square or a diamond and was presented 0.5° to the left or right (inside the array) or 4.2° (outside the array) from the center of the circular array. When the shape was presented inside the circular array, it was of 0.3°, while it was 0.9° when presented outside. The subjects were asked to report whether a square or diamond was present inside the circles. The other circles were either blank or filled with different geometric shapes, such as triangles and circles (see Figure 1B for example). Diamonds and squares were never displayed inside the circles simultaneously in the same trial, while four (Easy condition) or six (Hard condition) filled circles with shapes were evenly distributed over 320 trials. Additionally, in a counterbalance fashion, half of the trials were compatible (the figure outside the circular array matched the inside target shape), while the other half were incompatible (the figure outside the circular array did not match the inside target shape). The array was presented for 100 ms, followed by an unlimited amount of time to answer. Participants had to answer to continue the task. Once the answer was given, a 1000 ms fixation dot was displayed. Descriptions of the task and conditions can be found in Figure 1B. For additional details regarding the task, see Proksch and Bavelier (2002) and Green and Bavelier (2003).

#### Counting Task

The CT was implemented as described in Green and Bavelier (2003). In this task, a random number of squares was presented on the screen. Participants reported the number of squares that they observed. Squares (0.5° × 0.5°) were randomly displayed over a 10° × 10° square array centered on the screen. The set of squares were displayed for 100 ms, followed by the display of a visual fixation dot while awaiting the participants answer. The participants needed to respond to continue the task (Figure 1C). Once a response was given, the fixation dot was displayed for 1000 ms before the next trial. Task difficulty was modulated by presenting anywhere from 1 to 10 squares, which were presented in an even distribution across 200 trials.

During preliminary testing of the experimental procedure, we detected a cumulative effect on fingertip temperature that would reduce the comparability of the results within the tasks (for details, see “Results” Section). Additionally, the participants described the different tasks as differentially appealing, as they considered the CPT tedious and monotonous, while the CT was reported as fun or amusing. These differences conspired to directly compare the results obtained from the same task in pseudorandom order. Since our objective of employing many tasks was to examine whether thermoregulatory variables could predict more than one attentional task performance and that randomizing task order would importantly increase the sample size needed to detect such effects, we decided to have all the subjects perform the tasks in a fixed order. Based on the participant’s reports and to assure task engagement, we ordered the tasks from the most monotonous to amusing in the following order: BT, CTP, FT and CT. This sequence enabled us to increase the comparability of the results within the tasks while decreasing the comparability between tasks. For this reason, we include no conclusions based on comparisons between task results.

### Temperature Measurements and Manipulation

One hour before the arrival of the participant, the room temperature was set at a constant temperature between 18 and 26°C. The temperature was set among participants to acquire a continuous environmental sampling from this range. The temperature range was chosen based on previous room/environmental temperature studies (Steinmetz and Mussweiler, 2011; Cheema and Patrick, 2012; Huang et al., 2014; Schilder et al., 2014), and we intended to find an optimal room temperature rather than a safety range. Every participant was subjected to the same temperature throughout the entire experimental session. After the arrival of the participant, an additional 30 min were spent on the description of the experiment and reading/signing the informed consent forms. Peripheral vasoconstriction for thermoregulation is expected to plateau approximately 15–20 min after an environmental temperature change (Charkoudian, 2003), followed in almost identical fashion by skin temperature (Iampietro, 1971). Therefore, the time in the lab before the experiment was considered sufficient for acclimation.

To measure all four temperatures (tympanic, forehead, fingertip and room temperature), we built a measurement device using three Dallas DS18B20 thermometers, an infrared thermometer MLX90614, and an Arduino UNO board. We used the Arduino UNO for sampling the Dallas DS18B20 thermometers using a 1-wire interface, while the infrared MLX90614 thermometer was independently sampled with the same Arduino. Data sampled by the Arduino was sent *in situ* by a serial port to a computer. An *ad hoc* software developed in Python received the information from the Arduino and directly wrote it to the hard drive. This software also displayed a 3 min window presenting all four temperature measurements. This helped assure minimum room temperature variation throughout the experimental session. We also positioned two photoresistors in the two bottom corners of the screen. The tasks displayed a black or white square in these corners at programmed times, allowing us to synchronize the tasks with temperature measurements in time. These photoresistors were also sampled with the same Arduino board and recorded by the same Python *ad hoc* software.

The infrared thermometer was used to measure tympanic temperature, while the Dallas thermometers were used to measure the room, forehead and fingertip temperatures. The infrared thermometer was placed in the same fashion as an earphone in the left ear. We allowed the reading to reach a plateau measurement of approximately 36°C. Fingertip temperature was measured by fixating the thermometer to the left ring finger using adhesive tape. Participants were instructed to keep their left hand still and open to avoid contacting the thermometer with other surfaces. Forehead temperature was measured by fixating the thermometer above the participants left eye using adhesive tape. Finally, room temperature was measured by positioning a thermometer 40 cm in front and 20 cm to the left of the participant. The thermometer was lifted 5 cm from the desk in front of the participant, ensuring that contact was made only with the room air. In all experimental sessions, we observed a standard deviation of room temperature in the range of 0.14°C to 0.59°C, with an average of 0.3°C. Therefore, the variability of room temperature within the sessions was comparable among the participants.

### Data Analysis

Data analysis was designed to achieve the main goal of predicting performance based on body temperatures. Nonetheless, to do so, we first had to establish the existence of a cumulative effect to define a proper analytic strategy. Once we established the cumulative effect, we defined an analytical strategy to predict performance based on body temperature. For establishing that cumulative effects were taking place, we conducted a one-way repeated-measures ANOVA to compare the effects of the tasks (baseline, CPT, flanker, counting) on the environmental, forehead, fingertip and tympanic temperatures. When the sphericity assumption was not met, we corrected the p-value based on the Greenhouse-Geisser method. We reported the Greenhouse-Geisser epsilon (GGe), followed by the corrected *p*-value (p[GG]). For all ANOVA models, the effect size was reported using generalized eta-squared and denoted as $\eta_{\text{G}}^{2}$ (Bakeman, 2005). We maintained the ANOVA approach when the normality assumption was not met, as ANOVA is robust under such scenarios (Schmider et al., 2010). Nonetheless, we still reported if the normality of the dependent variable was violated using the Shapiro-Wilks test. Based on preliminary results, we also included two more variables. The first, ΔFingerT° was defined as:

(1) ΔFingerT°=Final Finger Temperature−Initial Finger Temperature

being the final finger temperature of the last 72 samples (approximately 1 min) obtained from the fingertip thermometer at the end of one of the four tasks. We considered the initial finger temperature as the value obtained from the first 72 samples (approximately 1 min) from the same task. The initial finger temperature did not include instruction time and was restricted to the beginning of the task. The second variable included was the instruction finger slope. This variable was obtained as the slope of finger temperature against time of one instruction period obtained from a linear regression calculation. We were unable to develop the same procedure described in ΔFingerT° due to high variability in the time spent in the instruction period.

For the main goal, predicting performance, we used multiple regression analysis. To perform this analysis, we used performance in the different tasks (excluding baseline) as dependent variables and all temperatures recorded plus the ΔFingerT° and instruction finger slope as regressors. The variables analyzed for each task were those most widely reported as performance markers. We also reported whether the dependent variable and residual data passed the assumption of normality, as well as homoscedasticity. Homoscedasticity was evaluated with the Non-constant variance score test, and for normality, we used the Shapiro-Wilk test. No normalization was used for any variable to maintain the meaning of the ranges observed. Finally, considering the obvious relationship among the independent variables, we tested collinearity for all the models using the variance inflation factor (VIF). Linear models presenting VIFs higher than three were then revised by checking each regressor alone and in combination, retaining the regressors that explained more variance (R squared-based) while also presenting VIFs below 3. This value was selected as a conservative value for the assumption of independence between independent variables (García et al., 2015).

For the CPT task, we used the Go and False Alarm reaction times and the percentage of correct answers as dependent variables. For the FT, we used incompatible minus compatible reaction times for the Easy and Hard conditions. Finally, for the CT, we first ran one multiple regression model with the error percentage as the dependent variable for each set of squares displayed (from one to ten). Based on these results, we unified the model by including the number of squares as an interaction. As already mentioned above, the independent variables (or regressors) were environmental temperature, fingertip temperature, forehead temperature, tympanic temperature, instruction finger slope and ΔFingerT°.

To visualize the stability of the participant’s room temperature and how consistent the fingertip temperature changed within each participant, we used an average centering (of each task) by subject. This allowed us to compare differences across experimental sessions by removing the differences in room temperature conditions as well as the differences in the starting fingertip temperatures. Additionally, as we wanted to present the real range at which the temperatures were measured, we added the grand average (of the sample). Thus, the figures are informative of the temperature ranges obtained. This procedure is summarized by the following equation,

(2) $$T_{\text{ij}}^{\text{°}}=(x_{\text{ij}}-x_{\text{j}})+x_{\text{g}}$$

where *x*_ij_ is an individual observation i of the temperature of a particular participant j, *x*_j_ is the participant’s j average temperature, *x*_g_ is the grand average temperature for all j participants and *T*°_ij_ is the centered temperature in an observation i and participant j. The same equation was used for centering the room, forehead, tympanic and fingertip temperatures. Equation 2 was used only for visualization purposes, while data were included without this centering for analysis.

As different participants took different amounts of time to complete the tasks or read the instructions, we also calculated a representative time length of each task/instruction using the following normalization scheme: we estimated a bin size for each task/instruction by calculating the average length of the group of participants. Each individual’s task-related temperature vector was then interpolated in that common space, taking the average length of the CT as the one with the norm equal to one. The CT was arbitrarily selected as the reference only to maintain the length ratio between tasks. Selecting another task as the reference would have led to the same results.

### Software

For all statistical analysis, we used R-project (R Core Team, 2015) with the following packages: lattice (Sarkar, 2008), ez (Lawrence, 2016) and car (Fox and Weisberg, 2011). Data processing and figures were developed with Python 2.7.1 and Anaconda 2.4.1 (Python Core Team, 2015) using the following packages: Pandas (McKinney, 2010), Matplotlib (Hunter, 2007) and rpy2 (rpy2 Core Team, 2015). All analyses and data processing were kept in a Jupyter Notebook (Kluyver et al., 2016).

## Results

### Tasks and Cumulative Differences

**Figure 2:**
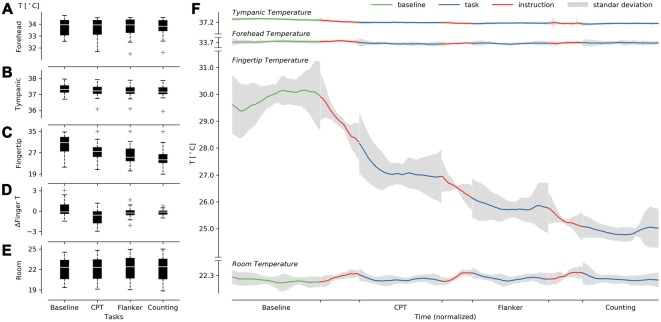
Description of the cumulative effect of the tasks on fingertip temperature under constant room temperature. **(A–C)** Present boxplots in successively performed tasks for forehead **(A)**, tympanic **(B)**, fingertip **(C)**, ΔFingerT° **(D)**, and room temperature **(E)**. On the right **(F)**, we present forehead, tympanic, fingertip, and room temperature averages centered (see Equation 2) over the normalized time (see “Materials and Methods” Section). The temperatures were centered to visualize the consistency in fingertip temperature reduction and room temperature stability across participants.

We tried to determine whether changes in environmental, tympanic, forehead, or fingertip temperatures could be seen when compared against a baseline. No significant differences were detected between the four tasks (baseline, CPT, flanker and counting) for room temperature (*F*_(3,54)_ = 0.88, GGe = 0.505, p[GG] = 0.396, $\eta_{\text{G}}^{2}$ = 0.0004; normality: *W* = 0.95, *p* = 0.007; Figure 2E), forehead temperature (*F*_(3,54)_ = 0.67, GGe = 0.447, p[GG] = 0.460, $\eta_{\text{G}}^{2}$ = 0.001; normality: *W* = 0.91, *p* = 0.0001; Figure 2A) and instruction finger slope (*F*_(2,36)_ = 0.30, *p* = 0.73, $\eta_{\text{G}}^{2}$ = 0.011; normality: *W* = 0.89, *p* = 0.0001). Instruction finger slope did not consider the BT, as the instructions were too brief in some cases to obtain the slope. Therefore, ANOVA results only contrasted the differences among attentional tasks. However, we did detect significant effects of tympanic temperature (*F*_(3,54)_ = 9.55, GGe = 0.48, p[GG] = 0.001, $\eta_{\text{G}}^{2}$ = 0.02; normality: *W* = 0.94, *p* = 0.001; Figure 2B), fingertip temperature (*F*_(3,54)_ = 35.04, GGe = 0.48, p[GG] = 2.79*e*-07, $\eta_{\text{G}}^{2}$ = 0.20; normality: *W* = 0.97, *p* = 0.169; Figure 2C) and ΔFingerT° (*F*_(3,54)_ = 5.8, GGe = 0.68, p[GG] = 0.005, $\eta_{\text{G}}^{2}$ = 0.19; normality: *W* = 0.95, *p* = 0.007; Figure 2D). When reviewing *post hoc* analysis using the Holm-Sidak test, no significant results were found for tympanic temperature, probably due to the lack of normality and the observed low effect size. However, we did find significant *post hoc* results for the fingertip temperature, specifically between the baseline (*M* = 29.88, SD = 3.69) and both the flanker (Holm: *M* = 25.81, SD = 3.87, *p* = 0.006) and CTs (Holm: *M* = 24.91, SD = 3.67, *p* = 0.0006). As shown in Figures 2C,D,F, fingertip temperature reductions during each instruction and during the tasks explains the results obtained. This confirmed a cumulative effect of the tasks on fingertip temperature throughout the experiment starting with the CPT task. Interestingly, the baseline did not exhibit a decrement in fingertip temperature. The reduction in temperature was strongly triggered by the instructions, while a smaller decrement in temperature was found during the task execution. When reviewing the *post hoc* ΔFingerT° results, the only significant result was observed between the CPT (*M* = −0.871, SD = 1.17) and baseline (Holm: *M* = 0.407, SD = 1.19 *p* = 0.0006). Nonetheless, the attentional task distributions were mostly below zero (fingertip cooling) except for the baseline, which presented a distribution mostly above zero (fingertip warming; Figure 2D). Since our sample size was rather small, we suggest that the negative results observed when contrasting the BT with the flanker and CTs may be false negatives due to low statistical power.

Overall, the results support a cumulative effect throughout the experiment, depicted as a reduction in finger temperature triggered by attentional tasks. We could see a constant, or even incremental, fingertip temperature as a general pattern only during the BT. Room temperature was stable throughout all the sessions, as depicted in Figure 2E and supported by ANOVA results, suggesting that the reduction in finger temperature was not due to room temperature change. It is worth noting that some participants presented increases in fingertip temperature within a task. This last phenomenon allowed us to test whether this decrease or increase in fingertip temperature might indicate the performance obtained during the task using ΔFingerT°.

### Performance Prediction

Once we confirmed that a cumulative reduction in fingertip temperature was taking place and that ΔFingerT° was mostly positive during baseline rather than negative during the remaining tasks, we examined whether we could predict performance for all three attentional tasks based on temperature variables. To that effect, we tested all the different temperature variables measured and obtained while leaving only those that were significant. In the case of collinearity, we discarded the variable or set of variables that presented the highest *R*^2^ and exhibited a VIF lower than 3 (for more details see “Materials and Methods” Section).

**Figure 3:**
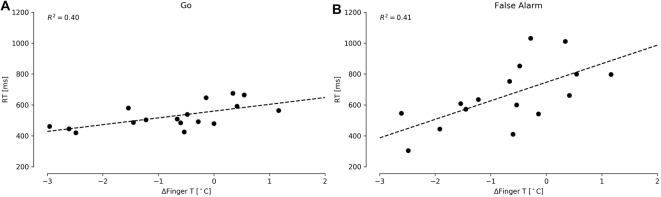
Reaction times for the CPT Go **(A)** and False Alarm **(B)** conditions under different ΔFingerT° values. The dashed lines depict linear regression model prediction.

We first examined whether the temperature data could predict performance in the CPT, specifically testing the accuracy and reaction answer times in the Go and False Alarm conditions. We did not find significant predictors for accuracy. However, we found that ΔFingerT° did predict the Go reaction times (*F*_(1,15)_ = 10.17, *p* = 0.006, *R*^2^ = 0.40; normality DV: *W* = 0.97, *p* = 0.83; normality residuals: *W* = 0.94, *p* = 0.42; homoscedasticity: *χ*^2^ = 1.31, *df* = 1, *p* = 0.25) and False Alarms (*F*_(1,14)_ = 9.79, *p* = 0.007, R2 = 0.41; normality DV: *W* = 0.92, *p* = 0.18; normality residuals: *W* = 0.98, *p* = 0.99; homoscedasticity: *χ*^2^ = 0.53, *df* = 1, *p* = 0.46). In these two models, only ΔFingerT° was a significant predictor of reaction time, specifically predicting that a decrease in fingertip temperature of one Celsius degree during the task would be associated with a reduction of 43.19 ms (±13.77) in the Go condition reaction time (*β* = 43.91, *t* = 3.18, *p* = 0.006) and 119.98 ms (±38.33) in the False Alarm condition reaction time (*β* = 119.98, *t* = 3.13, *p* = 0.007). As is depicted in Figures 3A,B, CPT reaction times could be predicted using fingertip temperature decreases/increases. Importantly, fingertip temperature did not predict reaction times and instead predicted only changes in reaction time (ΔFingerT°).

**Figure 4:**
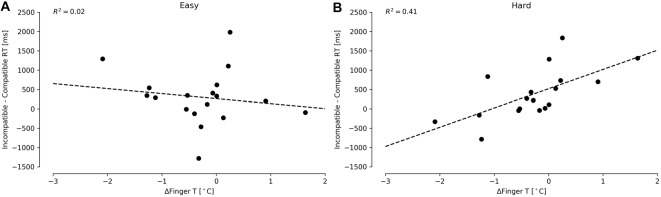
Differences between incompatible and compatible reaction times in the FT for Easy **(A)** and Hard **(B)** conditions under different ΔFingerT° values. The Easy **(A)** condition does not present a significant prediction, while the Hard condition **(B)** does. The dashed lines depict linear regression model prediction.

For the FT, we focused on the difference in reaction times of incompatible and compatible conditions, separately analyzing the Easy and Hard conditions according to Proksch and Bavelier (2002) and Green and Bavelier (2003). The increment in the difference between incompatible and compatible reaction times conditions was interpreted as resiliency to distractors. Higher values depicted higher difficulties in ignoring distractors. We detected a considerable distribution of outliers by subject, presenting an average of approximately 600 ms, with observations up to 10 s. For this reason, we decided to remove outliers using criteria based on two-standard deviations (based on grand average and grand standard deviation). We found only one significant predictor for the Hard condition: ΔFingerT° (Figure 4A; *F*_(1,16)_ = 11.5, *p* = 0.003, *R*^2^ = 0.41; normality DV: *W* = 0.96, *p* = 0.67; normality residuals: *W* = 0.88, *p* = 0.03; homoscedasticity: *χ*^2^ = 0.007, *df* = 1, *p* = 0.93). No significant regressor was found when predicting the Easy condition, as a low fit was obtained when using ΔFingerT° (Figure 4B; *F*_(1,16)_ = 0.38, *p* = 0.54, *R*^2^ = 0.02; normality DV: *W* = 0.94, *p* = 0.38; normality residuals: *W* = 0.93, *p* = 0.20; homoscedasticity: *χ*^2^ = 0.15, *df* = 1, *p* = 0.69). Specifically, the model related to the Hard condition predicted an increase of 497.9 ms (±146.9) in the difference between the incompatible and compatible conditions by each increase of one Celsius degree in ΔFingerT° (*β* = 497.9, *t* = 3.39, *p* = 0.003). As such, increasing ΔFingerT° was associated with more difficulty in ignoring distractors, while the opposite ΔFingerT° was decreasing (hand cooling). These results were robust enough, as using three standard deviation criteria for removing the outliers led to the same results, albeit with lower fits (data not shown). The fact that no significant prediction was made for the Easy condition supports that this effect is only observable when the task is sufficiently difficult.

**Figure 5:**
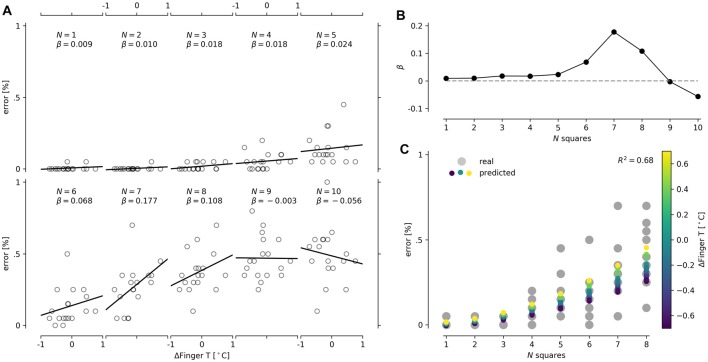
Depiction of interaction between the number of squares presented in the CT and ΔFingerT°. Scatter plots for each number of squares (N) presented in the CT are shown on the left **(A)**. We also include a solid line for linear regression and its slope (β) in each scatter plot. For better visualization of the change in slope (the interaction), a line plot of the slopes (β) and the number of squares is presented **(B)**. Finally, the prediction of the linear model containing the interaction between the number of squares (*x*-axis) and ΔFingerT° (color scale) was plotted with the actual data obtained (gray dots; **C**).

The last conclusion was straightforward to retest in the CT, as increasing the number of squares increased the difficulty of the task. We found that the best and only predictor of performance in the CT was ΔFingerT°. Particularly, we observed that the percentage of error when counting the squares was independent of ΔFingerT° at a low number of squares, while progressively higher slopes in the relationship between error percentage and ΔFingerT° were achieved as the number of squares increased (see Figures 5A,B). Nonetheless, at high difficulties (8, 9 and 10 squares), we observed a quick decrement in the slope of this relation. This finding suggests that a task shows a relation between ΔFingerT° and error percentage only when it is not too easy or too hard. To model this complex relationship in one linear model, we included an interaction term between ΔFingerT° and the number of squares. However, this was not enough to model the decrement of slopes from eight or more squares (see Figure 5B). Therefore, we decided to only model squares 1 through 7. When modeling this interaction as a linear change, we obtained significant results (*F*_(3,122)_ = 36.2, *p* = 2.2*e*-16, *R*^2^ = 0.47; normality DV: *W* = 0.84, *p* = 0.1.2*e*-12; normality residuals: *W* = 0.87, *p* = 7.5*e*-09; homoscedasticity: *χ*^2^ = 47.02, *df* = 1, *p* = 7.0*e*-12); however, is important to note that the change in slope had exponential growth. For the sake of simplicity, we modeled this exponential slope growth with a quadratic function by introducing the number of squares squared (#squares^2^). This strategy also led to significant results (*F*_(3,122)_ = 44.34, *p* = 2.2*e*-16, *R*^2^ = 0.52; normality DV: *W* = 0.84, *p* = 0.1.2*e*-12; normality residuals: *W* = 0.83, *p* = 1.8*e*-10; homoscedasticity: *χ*^2^ = 55.5, *df* = 1, *p* = 9.2*e*-14). In an attempt to improve the model’s assumptions (previous models presented heteroscedasticity), we decided to fix the intercept to zero, as zero squares should have zero errors. After applying this strategy, we also obtained significant results (*F*_(3,123)_ = 83.92, *p* = 2.2*e*-16, *R*^2^ = 0.67; normality DV: *W* = 0.84, *p* = 0.1.2*e*-12; normality residuals: *W* = 0.82, *p* = 4.1*e*-11; homoscedasticity: *χ*^2^ = 54.6, *df* = 1, *p* = 1.4*e*-13), but the model assumption was not improved. The last model, seen in Figure 5C, specifically suggests that reductions in finger temperature (negative values of ΔFingerT°) predict a decrease in the percentage of errors only at a high number of squares (6–7) (ΔFingerT°: *β* = −0.002, *t* = −0.069, *p* = 0.94; squared number of squares: β = −0.005, *t* = 15.8, *p* = 2*e*-16; interaction: *β* = 0.002, *t* = 2.23, *p* = 0.027). Overall, these results support that performance in the CT and ΔFingerT° are related only when the task is not extremely easy or hard. Importantly, the relation is robust enough to be detected using different linear modeling approaches despite the fact that no linear regression met homoscedasticity assumptions.

In summary, ΔFingerT° was the best predictor of performance in all the tasks analyzed. Better predictions were achieved when the tasks were not extremely easy or hard. This finding suggests that thermoregulatory related variables, specifically ΔFingerT°, could predict alertness, as performances in all the tasks were predicted by ΔFingerT°. Importantly, ΔFingerT° change was triggered by the tasks and even the task instructions, but not by the baseline. Nevertheless, it is worth noting that changes in fingertip temperature were unlikely due to thermoregulatory processes, as the room temperature was kept virtually constant (Figures 2B,C) and the participants had sufficient time to acclimatize (see “Materials and Methods” Section).

## Discussion

We selected three attentional tasks to predict performance using peripheral thermoregulatory variables: a CPT to measure the ability of maintaining attention over time (Huang-Pollock et al., 2012), a FT to measure the ability to ignore distractors (Proksch and Bavelier, 2002; Green and Bavelier, 2003), and a CT to measure the availability of attentional resources (Green and Bavelier, 2003). In all three tasks, we could predict performance using reaction times and accuracy within a 18–24°C range when the task was not extremely easy or hard. This strategy allowed the prediction of performance regardless of the task type and complexity.

### Cumulative Effect

The results presented herein showed how only sitting and relaxing (baseline) do not trigger conspicuous changes in fingertip temperature, while all three attentional tasks triggered a reduction in its temperature as early as when the instructions were read. Participants were acclimated before starting these tasks, suggesting that an autonomic response is triggered by an alert state that shares thermoregulatory mechanisms. Fingertip temperature reductions have a cumulative pattern that decreases with time, as they were higher in the beginning and got smaller throughout the experiment. In all the tasks tested, bigger decrements (high negative values of ΔFingerT°) within the tasks were associated with better performance. As such, longer exposures should have equated with worse performance, which is apparently consistent with previous studies based on environmental temperature (Hancock, 1986; Pilcher et al., 2002; Hancock and Vasmatzidis, 2003; Hancock et al., 2007; López-Sánchez and Hancock, 2017). However, this can also be explained by an autonomic response related to an arousal that shares vasoconstriction/vasodilation thermoregulatory mechanisms. Additionally, our results suggest that reductions in ΔFingerT° ranges throughout the tasks are produced by the bottom environmental temperature limit. Vasoconstriction in the fingertip likely reduces the amount of heat that arrives at the limbs, thereby reducing fingertip temperature (Charkoudian, 2003, 2010; Johnson and Kellogg, 2010). At the same time, fingertip temperature cannot be reduced beyond that of the environmental temperature, even if vasoconstriction continues to increase. As fingertip temperature gets closer to that of the environment, reductions should get smaller and slower as the thermal gradient decreases. The cumulative process discussed here is the main reason why we decided not to randomize the task orders. Since fingertip temperature reduction size depends on the order of the task, a larger sample size would have been required to generate enough statistical power to compare the same task in different orders when predicting with ΔFingerT°. We are aware that this may constitute a limitation of the study, but it enabled us to better understand whether ΔFingerT° can predict performance (in this circumstance). Estimating the importance of the ΔFingerT° magnitude when predicting task performance is not possible with these data. Since we maintained similar reductions for each task (by fixing the order of the tasks), the CTs presented a smaller ΔFingerT° range than that of the and FT. Nonetheless, the ΔFingerT° sign seems to be robust enough to inform aroused (when negative) or inattentive (when positive) states.

### Performance and ΔFingerT°

Changes in fingertip temperature (direction and module) were large enough to predict performance in all three of the tasks. All the other measured temperatures (tympanic, forehead, fingertip and room) were also tested as predictors, and they were not significant in the presence of ΔFingerT°. However, why only the fingertip temperature changed remains unclear. Notably, vasoconstriction/dilation occurs mainly in the limbs, as hands and feet have a higher surface to volume ratio. This means that hands and feet will have stronger thermal oscillations due to autonomic activity and environmental temperature compared to forehead temperature (Cheung, 2015). Finally, the stable maintenance of tympanic temperature strongly suggests that this is a vasoconstriction/dilation phenomenon that is not necessarily directly tied to thermoregulation. Notably, fingertip temperature reduction during the reading of the instructions (measured using the instruction finger slope) did not predict posterior performance within the task. We speculate that the fingertip reduction during the instructions accounts only for engagement with the instructions and thus may not directly predict further performance in the task. The fact that this response occurred as early as the instructions suggests that this autonomic response is a preparatory answer for the upcoming task. Arousal is known to lead to major autonomic modulations (Kreibig, 2010) that are also related to other aspects, such as motor preparation (Drabant et al., 2011). However, this preparatory answer seems to be a general response that does not necessarily imply better performance in the upcoming task. When considering in-task fingertip temperature modulations, ΔFingerT° predicted the reaction times for CPT in the Go and FA conditions. This means that ΔFingerT° predicts speed but not precision in CPT, unlike in the counting and FTs in which precisions were predicted. Sustained attention tasks had the speed vs. precision tradeoff that was not present in the flanker and CTs. Specifically, performance is modulated by the task instructions, changing the participant’s priorities and therefore modifying the results (Peebles and Bothell, 2004; Helton et al., 2009). Notably, giving the instruction to prioritize speed and precision equally leads to lower performance and increases variance (Seli et al., 2012). In our current research, we biased the instructions to prioritize speed, which explains why precision was compromised. We think that the speed-precision tradeoff and our current instructions made the participants use their attentional resources to improve speed but not precision. Altogether, these results support that ΔFingerT° was a predictor of alertness. Currently, most predictions to avoid accidents are “offline”, as safe work conditions are encouraged rather than warned against ongoing unsafe behaviors (Huting et al., 2016; Klerman et al., 2016). Some studies have shown that real-time warning is possible using eye tracking, skin conductance, electrocardiogram, and electroencephalography (Dahiphale and Rao, 2015; Larue et al., 2015). However, to our knowledge, no reports include finger temperature as a relevant real-time maker of alertness. Therefore, it is worth discussing its advantages and limitations. Problems with giving real-time warnings and safe work conditions include difficulty and workload, which are already known to be problems when dealing with thermal stress (Ramsey et al., 1983; Pilcher et al., 2002; Hancock et al., 2007). The FT and CTs allowed us to test how difficulty affected the prediction made by skin temperature. Our results supported previous findings (Hancock, 1986; Pilcher et al., 2002; Hancock et al., 2007) in that difficulty was critical for the proper prediction of performance by ΔFingerT°. However, our main goal was to find a thermoregulatory marker to bypass the problems associated with the task type, extension, complexity and difficulty. In fact, ΔFingerT° was not enough to bypass task difficulty only for the top and bottom performances. Naturally, when participants give correct or incorrect answers approximately 100% of the time, predicting performance using another variable is impossible since performance is constant. Nonetheless, if we accept that ΔFingerT° measures alertness, we could interpret a participant’s engagement despite his or her performance. We think that this is a limitation of the task (which can present the top and bottom effects) rather than a limitation of the ΔFingerT° prediction. This suggests that fingertip temperature can be used as an alertness marker to detect whether a participant is engaged or disengaged in a task, which is similar to what was previously shown in a study using finger temperature as a stress marker (Yamakoshi et al., 2007).

### Physiological Mechanisms of Temperature Modulation

We proposed using peripheral thermoregulatory variables to better estimate the effects of the widely reported environmental temperature effect on attention (Hancock, 1986; Pilcher et al., 2002; Hancock et al., 2007; Cheema and Patrick, 2012; Huang et al., 2014; López-Sánchez and Hancock, 2017) by including subjective thermoregulatory differences. Our experiment was designed to present constant values for all the temperatures for each participant to then compare temperatures among participants. Specifically, comparing different environmental temperatures (ranging between 18–24°C), presumably affecting body temperature, would lead to different attentional states. Our results showed that even in the thermoneutral zone and under acclimated conditions, peripheral vasoconstriction/dilation could be triggered for non-thermoregulatory reasons. As such, we proposed that the reduction in fingertip temperature triggered by a task is a light stress response that increases arousal for fulfilling the task. It is well known that thermal stress induces vasoconstriction/dilation (Charkoudian, 2003; Johnson and Kellogg, 2010), which changes hand and feet temperatures (Cheung, 2015). If we also consider that cognitive-demanding tasks produce vasoconstriction (Iani et al., 2004) and that subjective self-reported stress (non-thermal stress) ratings can also be followed with a similar ΔFingerT° strategy (Yamakoshi et al., 2007), peripheral vasoconstriction is also triggered by psychological phenomena. The vasoconstriction observed in this study, likely triggered by the tasks, may have important physiological implications. Peripheral vasoconstriction reduces body thermal conductivity, thus increasing internal body temperature. It is known that the brain increases its temperature during tasks (Abrams and Hammel, 1964; Shevelev, 1998; Kiyatkin et al., 2002; Zhu et al., 2009; Wang et al., 2014), which is attributed to brain-increased metabolism. However, our results suggest that this increase should be at least partially caused by peripheral vasoconstriction. Conversely, we did not find any change in body core temperature (as measured by tympanic temperature), suggesting that vasoconstriction is not enough to increase body temperature. Another explanation could be that most extra heat produced by brain metabolism plus the increase in body thermal isolation due to peripheral vasoconstriction is counteracted by cerebral blood flow. One of the most important mechanisms controlling brain temperature is cerebral blood flow (Zhu et al., 2009; Wang et al., 2014). If increased heat production and duration are not big and long enough, respectively, water in blood will capture the heat without increasing the body core temperature due to its specific heat (Sawka et al., 2011). We should expect that longer task durations might be associated with minor increases in body core temperature or that vasodilation (fingertip warm up) will dissipate heat. Under any circumstance, if environmental temperature increases too dramatically (>30°C), the alert driving vasoconstriction might counteract thermoregulatory mechanisms. Including the reduction in metabolic rate to reduce heat production (Seebacher, 2009) should have even worse effects on cognitive functions by also limiting brain activity. In fact, hyperthermia was previously reported to reduce cluster size activation in an fMRI study during an attentional task (Liu et al., 2013). As such, our results further highlight the need for properly controlling safety environmental temperature ranges during work as well as the duration of exposure to high temperatures. Despite its limitations, the present study marks the importance of using proper physiological research to understand psychological consequences. Our current results support that classic thermoregulatory mechanisms are triggered by psychological processes, increasing both complexity and strategy to couple with thermal stress.

### Perspective and Possible Applications

The work presented herein suggest that peripheral body temperature can be valuable for predicting the alertness and performance of participants. Furthermore, our results suggest that utilizing the raw fingertip temperature value not only measures thermoregulatory acclimation but also alertness when using ΔFingerT°. We expect that under harsher environments (environmental temperatures beyond the 18–24°C), raw fingertip temperature values can be important predictors of performance. However, caution is advised, as the effects of difficulty on our predictions narrow the possible applications of ΔFingerT° to only demanding tasks or untrained personnel. Additionally, the non-randomization of tasks restricts the extrapolation of our results, and further research is needed to test the robustness of how ΔFingerT° functions as a performance predictor, particularly in relation to its module rather than direction. Nonetheless, we believe that fingertip temperature and derived measurements can greatly help avoid accidents using real-time warnings.

## Author Contributions

The experimental design was developed by RCV and PEM, data collection was performed by RCV, and analyses were done by CM-L and RCV. All electrical *ad hoc* devices and software were developed by RCV. The manuscript was written by RCV and PEM.

## Conflict of Interest Statement

The authors declare that the research was conducted in the absence of any commercial or financial relationships that could be construed as a potential conflict of interest.
